# Distribution and transmission of *Mycobacterium tuberculosis* complex lineages among children in peri-urban Kampala, Uganda

**DOI:** 10.1186/s12887-015-0455-z

**Published:** 2015-09-30

**Authors:** Eddie M. Wampande, Ezekiel Mupere, Devan Jaganath, Mary Nsereko, Harriet K. Mayanja, Kathleen Eisenach, W. Henry Boom, Sebastien Gagneux, Moses L. Joloba, For the Tuberculosis Research Unit (TBRU)

**Affiliations:** Department of Medical Microbiology, College of Health Sciences, Makerere University, P.O box 7072, Kampala, Uganda; Department of Pediatrics and Child Health College of Health Sciences, Makerere University, Kampala, Uganda; Uganda-Case Western Reserve University Research Collaboration, Kampala, Uganda; Department of Pathology, University of Arkansas for Medical Sciences, Little Rock, Arkansas USA; Tuberculosis Research Unit, School of Medicine, Case Western Reserve University and University Hospitals of Cleveland, Cleveland, Ohio USA; Department of Medical Parasitology and Infection Biology, Swiss Tropical and Public Health Institute, Basel, Switzerland; University of Basel, Basel, Switzerland; Department of Bio-molecular Resources and Biolab Sciences, College of Veterinary Medicine, Animal Resources and Bio Security, Makerere University, Kampala, Uganda; Department of Medicine College of Health Sciences, Makerere University, Kampala, Uganda; The Johns Hopkins School of medicine, Department of Pediatricss, Baltimore, USA

**Keywords:** Household, Childhood tuberculosis, Transmission, MTBC lineages

## Abstract

**Background:**

To gain insight into the transmission of tuberculosis (TB) in peri-urban Kampala-Uganda, we performed a household contact study using children as a surrogate for recent transmission of *Mycobacterium tuberculosis* (MTB). Using this approach, we sought to understand *M. tuberculosis* complex (MTBC) lineage diversity, distribution and how these relate to TB transmission to exposed children.

**Method:**

MTBC isolates from children aged ≤ 15 years, collected from 2002 to 2010 in a household-contact study, were analyzed using a LightCycler RT-PCR SNP genotyping assay (LRPS). The resultant genotypic data was used to determine associations between MTBC lineage and the children’s clinical and epidemiological characteristics.

**Results and discussion:**

Of the 761 children surveyed, 9 % (69/761) had culture-positive TB an estimate in the range of global childhood TB; of these 71 % (49/69) were infected with an MTBC strain of the “Uganda family”, 17 % (12/69) infected with MTBC lineage 4 strains other than MTBC Uganda family and 12 % (8/69) infected with MTBC lineage 3, thereby disproportionately causing TB in the study area. Overall the data showed no correlation between the MTBC lineages studied and transmission (OR = 0.304; *P*-value = 0.251; CI: 95 %; 0.039-2.326) using children a proxy for TB transmission.

**Conclusions:**

Our findings indicate that MTBC Uganda family strains are the main cause of TB in children in peri-urban Kampala. Furthermore, MTBC lineages did not differ in their transmissibility to children.

## Background

Inhalation of aerosols containing *Mycobacterium tuberculosis* (MTB) bacilli by susceptible hosts is the main route of transmission of tuberculosis (TB), a disease that causes high mortality and morbidity with an estimated 74,000-130,000 cases in children and 1.5 million deaths per annum in adults [1–4]. Under certain circumstances, TB can also be vertically transmitted, i.e. congenital TB [1, 5]. A number of risk factors have been associated with MTB transmission. For instance such factors may be host related such as HIV co-infection, age, sex [6, 7] [8]; some are environmental such as overcrowding [9]. Moreover, MTBC strains may differ in virulence and transmissibility [4, 10–13]. Two approaches have been used to define MTB transmission in a community. The first uses MTBC phylogenetic clustering to signify on-going transmission (recent transmission) and the presence of unique strains as indicators of reactivation of latent TB infections [4, 14, 15]. A second approach defines transmission through pediatric TB, since children progress faster from recent MTB infection to disease than adults, in part because of immune system immaturity resulting in rapid disease progression rather than latency [16–18].

Like any other Sub-Saharan country, Uganda has a diversity of MTBC lineages with the MTBC Uganda family, a sub-lineage of lineage 4 (Euro-American lineage) being the main cause of TB followed by MTBC lineage 4 non-Uganda (Euro American lineages other than Uganda family) and MTBC lineage 3 (East Africa India/Central Asian Strain) [13]. Studies on transmission dynamics of these MTBC lineages in Uganda are not well described, yet could shed light on the relative distribution and dominance of the MTB Uganda family. For instance, the wide spatial distribution and dominance of the Beijing genotype, a member of MTBC lineage 2 (East Asia lineage), has been associated with increased transmissibility relative to other MTBC lineages in certain geographical regions [10, 11, 19]. These studies were done in adults where it is difficult to ascertain when transmission occurred [15]. Children on the other hand, negate this impediment since primary active disease progression is common due to a less developed TB specific immunity. Thus children provide an alternative model for studying on-going transmission of MTB.

In this study, childhood TB was used as a proxy for transmission in an effort to understand why the MTBC Uganda family has been dominant in peri-urban Kampala over several decades [20]. We hypothesized that the MTB Uganda family is more transmissible than other MTBC lineages circulating in peri-urban Kampala. To test this hypothesis, MTBC isolates from children aged ≤ 15 years were collected from a household cohort conducted from 2002 to 2010 as described by Jaganath et al., [21] and analyzed to understand the MTBC lineage distribution among the pediatric TB cases. In addition we examined the association between MTBC lineages and certain epidemiological and clinical variables.

## Materials and methods

### Study area and sample collection

Children in contact with adults who had pulmonary TB in peri-urban Kampala were studied. The study considered MTBC isolates that were collected from children aged ≤ 15 years using a well characterized TB household contact study conducted in the Kawempe area of Kampala, the Kawempe Community Household study (KC) [20]. The KC study focused on TB and its risk factors among adult patients (≥18) and their household contacts. Culture positive TB patients were consecutively enrolled as index cases (patient who reported first at the TB clinic with pulmonary TB) and their housemates (contacts) whom had stayed in the index case household for at least 7 consecutive days during the 3 months prior to the diagnosis of TB. Household contacts were classified as co-prevalent cases if active TB was present at baseline or during three months of household follow-up and as incident cases if active TB developed after three months of follow-up.

At baseline, data of enrolled children, including age, sex, HIV status and presence of BCG scar (deltoid scar) were recorded. Clinical features such as persistent cough for more than 3 weeks, night sweats and hemoptysis were documented. The radiological features such as extent of lung involvement classified as (i) normal/moderate [mild lung disease], where lesions appeared as infiltrates of slight to moderate densities affecting a small portion of one or both lungs with no cavitation (ii) advanced/far advanced [advanced lung disease], where lung lesions were more extensive than in mild lung disease category with cavities ≤ 4 cm, were documented. Tuberculin skin test (TST) was performed by Mantoux method (where a positive TST was defined as an induration of ≥ 10 mm if a child was HIV negative and ≥ 5 years of age or ≥ 5 mm if a child was HIV positive or < 5 years of age). Sputum or gastric lavage samples collected at baseline were microscopically examined and processed for culture at the Joint Clinical Research Centre (JCRC) TB Laboratory in Kampala following standard procedures [22]. Isolates were confirmed as MTBC using the BACTEC® para-nitro-acetyl amino-hydroxy-propiophenone (NAP) susceptibility method [23] after 4 weeks and stored at – 80 °C in 7H9 broth supplemented with OADC and glycerol for future analysis. In this study, a total of 761 children from 351 households were enrolled. Of these, 69 (9 %) were culture positive and had their bacilli isolated and stored for future use. In an earlier study, a total of 1746 isolates from adult (>15 years) patients within the same cohort were genotyped as described [13].

### Genomic DNA extraction from stored MTBC isolates

A total of 69 isolates were stored in replicates at – 80 °C. Isolates corresponding to an individual child were selected for genotyping. To extract DNA, the selected isolates were thawed overnight at – 20 °C and later at room temperature for 12 hours. The vials were centrifuged at 15,000 g for 30 min and the pellet washed twice with 500 μl of Qiagen PCR-grade water. The final pellet was re-suspended in 100 μl of Qiagen PCR-water, heated at 95 °C for 30 minutes to kill and lyse the bacilli and later sonicated for 15 min at room temperature. The extracted crude genomic DNA in the supernatant was recovered by centrifugation at 15,000 g for 30 min; the latter was used immediately in the real time PCR (RT-PCR) assay or stored at −20 ° C for future use.

### Genotyping the MTBC isolates from children

To ascertain the MTBC lineage to the 69 isolates, three single nucleotide polymorphism (SNP) markers were used. These are specific for identifying MTBC Uganda family (MTB L4-U), MTBC Lineage 4 and MTBC Lineage 3 (MTBC L3). Corresponding primers and probes were used in a LightCycler real time PCR SNP assay (LRPS) to identify the MTBC lineages on the basis of differences in melting temperature (Tm) as described by Wampande et al. [13]. In all the assays, we used MTB L4-U genomic DNA from our laboratory, H37Rv genomic DNA (Lineage 4) and MTB Lineage 3 (Central Asian strain) genomic DNA (Courtesy of Mark Nicol) as positive control DNA.

### Statistical analysis

The baseline characteristics of children with TB were compared with MTBC lineage using the chi-square test for binary data. A series of univariate and multivariable logistic regression models were fitted to evaluate the relationship between MTBC lineage (main exposure variable) and extent of lung involvement of TB disease (either Normal/moderate [mild lung disease]; or advanced/far advanced [advanced lung disease] on chest radiograph as the outcome [24]. The extent of lung involvement i.e. normal/moderate (mild lung disease) or advanced/far advanced (advanced lung disease) was used as a measure of disease severity and hence transmission [10, 12, 25]. The covariates age, sex, HIV status, presence of BCG scar, night sweats, hemoptysis, tuberculin skin test, and cough were used as adjusters. All analyses were performed using STATA version 12.

### Ethical consideration

The institutional review boards and ethics committees at Case Western Reserve University, Makerere University, Uganda AIDS Research Council, and the Uganda National Council for Science and Technology approved the study protocols. Patients/guardians gave written consent for the children and children aged ≥ 8 years provided additional assent. Pre- and post-HIV test counseling was provided.

## Results

### Characteristics of children with culture positive tuberculosis

A total of 761 children (from 351 household) were surveyed from 2002 to 2010 in a household study. Of these, 69 (9 %) children (from 62 household) were culture positive, this finding is similar to earlier studies carried out in the same study cohort [21]. Seventy-one percent (49/69) were children aged up to 5 years, 54 % (37/69) were females, 14 % (9/66) were HIV positive, 67 % (46/69) had a BCG scar, 12 % (8/69) reported signs of night sweating, 29 % (19/65) showed advanced lung disease on radiography, 42 % (29/69) had cough, 62 % (41/66) had a positive tuberculin skin test and 85 % (47/55) were smear negative (Table 1). Furthermore, 91 % (59/62) had no cavitation, and 97 % (67/69) also had no hemoptysis.

**Table 1:** Distribution of MTBC family among different pediatric TB risk factors

Variable	Category	Patients characteristics (N = 69)	MTB Uganda family (n = 49)	MTB non-Uganda (n = 20)	*P*-value
		(n, %)			
Age^1^	0-2years	30(43)	19(39)	11(55)	0.230
	2-5years	19(28)	13(27)	6(30)	
	5 above	20(29)	17(35)	3(15)	
Sex^2^	Female	37(54)	27(55)	10(50)	0.451
	Male	32(46)	22(45)	10(50)	
HIV status^3^	Negative	57(86)	40(85)	17(89)	0.489
	Positive	9(14)	7(15)	2(11)	
BCG scar^4^	Absent	23(33)	18(37)	15(75)	0.259
	Present	46(67)	31(63)	5(25)	
Night sweats^5^	Absent	61(88)	42(86)	19(95)	0.259
	Present	8(12)	7(14)	1(5)	
Extent of lung involvement^6^	Normal/Moderate	46(71)	36(78)	10(53)	0.041
	Advanced/Far Advanced	19(29)	10 (22)	9 (47)	
Cough^7^	No	40(58)	32(65)	8(40)	0.048
	Yes	29(42)	17(35)	12(60)	
Tuberculin skin test^8^	Negative	25(38)	21(45)	4(21)	0.063
	Positive	41(62)	26(55)	15(79)	
Smear status^9^	Negative	47(85)	30(79)	17(100)	0.041
	Positive	8(15)	8(21)	0(0)	

1,2,4,5,7 =Age, Sex, BCG scar, Night sweats and Cough missing 0 values 3 = HIV status missing 3 values, 6 =  extent of lung involvement missing 4 values, 8 = tuberculin skin test missing 3 values and 9 = smear status missing 14 values

### MTBC lineages infecting children in peri-urban Kampala

All 69 MTB isolates corresponding to an individual child were genotyped using LRPS as described by Wampande et al. [13]. Overall, 71 % (49/69) were MTBC L4-U (MTBC Uganda family), 17 % (12/69) MTBC L4-NU (Lineage 4 strains other than Uganda family) and 12 % (8/69) Linage 3 (MTBC L3). This data was comparable with MTBC lineages in the adult (≥18 years) patients published by Wampande et al. [13] from the same study cohort. Given that finding, further analysis was done to seek for possible association between MTBC lineages and the available clinical and epidemiological variables.

The additional data analysis (this analysis has combined MTB L4-NU and MTB L3 in one group: from now onwards this shall be referred to as MTBC non-Uganda) showed that children infected with MTBC non-Uganda lineage were more likely to have cough (*P* = 0.048) and advanced extent (Advance/far advanced) of lung involvement (P = 0.041) compared to those infected with MTBC Uganda family. Children infected with MTBC Uganda were more likely to have smear-positive TB (*P* = 0.041). No significant differences (*P* > 0.05) were observed between MTBC lineages with respect to age, sex, HIV/TB co-infection, presence of a BCG scar, night sweats and tuberculin skin test status (Table 1).

### Risk factors associated with advanced lung disease in children

Since advanced lung disease is positively correlated with severity of disease and hence a measure of transmissibility [10–12, 25, 26], we looked for differences in transmissibility among MTBC lineages, using advanced extent of lung involvement (mild or advanced lung disease) as the outcome in the logistic model. Multivariate analysis was performed to look for risk factors associated with advanced lung disease while using MTBC lineage as the main predictor. After adjusting for age, sex, HIV status, presence or absence of BCG scar, a child having or not having night sweats, children who cough and those who do not, data showed that the odds of a child having advanced lung disease did not differ significantly (OR = 0.304, CI: 95 %; 0.039-2.326, P = 0.251) between MTBC lineages and no other risk factor was associated with advanced lung disease (Table 2).

**Table 2:** Multivariate analysis for factors associated with advanced lung disease among 69 pediatric TB cases

	Category	Crude value		Adjusted value	
		OR; (95 % CI)	*P* values	OR (95 % CI)	*P* values
MTB genotypes	MTB Non-Uganda	1	0.043	1	0.251
	MTB Uganda	0.308 (0.099-0.966)		0.304 (0.039-2.326)	
Age	≤5 years	1	0.081	1	0.059
	>5 years	0.243(0.049-1.192)		0.065 (0.004-1.111),	
Sex	Female	1	0.900	1	0.677
	Male	1.071 (0.367-3.128)		1.405 (0.283-6.969),	
HIV status	Negative	1	0.977	1	0.307
	Positive	0.975 (0.171-5.555)		0.242 (0.0158-3.695)	
BCG Scar	Absent	1	0.219	1	0.307
	Present	2.198 (0.627-7.711),		1.075 (0.202-5.715)	
Night sweats	No	1	0.102	1	0.049
	Yes	3.822 (0.765-19.09)		20.809 (1.013-427-229)	
Cough	No	1	0.246	1	0.174
	Yes	1.895(0.643-5.589)		0.231(0.279-1.913)	
Tuberculin skin test	Negative	1	0.071	1	0.148
	Positive	3.150(0.906-10.953)		5.140 (0.558-47.322)	

## Discussion

In this study, genotypic data of MTBC isolates in combination with epidemiological and clinical data from children (≤15 years) was used to describe on-going TB transmission in peri-urban Kampala. The findings indicate that 9 % of children in peri-urban Kampala in this TB household contact study had culture confirmed TB and presented with paucibacillary TB, no cavitation and no hemoptysis as described by others [4, 16, 27, 28]. The current study had more patients analyzed compared to those described by Jaganath et al. [21] this is as a result a longer follow-up. However the TB prevalence between the 2 studies did not differ significantly. Moreover, this data found cough and extent of lung involvement significantly associated with MTB lineages but this disappeared in the multivariate analysis [29, 30]. The distribution of MTBC lineages in peri-urban Kampala between adults and children was similar, with MTBC L4-U as the most dominant, followed by MTBC L4-NU and MTBC L3. MTBC lineages did not differ in capacity to transmit or cause advanced lung disease in children.

Our data shows 9 % (69/761) childhood TB. Studies elsewhere demonstrate 1 % - 4 % using smear positive cases [4, 31, 32], contact investigation studies estimated a prevalence of 7 % -10 % [21, 33, 34] in a high TB burden setting and 5 % in low-burdened countries [32, 35]. Current global childhood TB estimates range from 10–15 % [35, 36], which is close to our findings.

The MTBC lineage distribution pattern in children and adults was similar consistent with adult to child TB transmission in a household. A related study in peri-urban Kampala demonstrated transmission between members of the same residence since they had similar strains [37]. 77 % (48/62) of the residences showed children infected with the same MTBC lineages as the adults, the other 23 % showing heterogeneous MTBC lineages suggesting community transmission from adult to children. Nevertheless, we cannot rule out the possibility that some children acquired more than one MTBC lineage from the adults. Indeed, similar studies in the study area showed that 7.1 % of the patients were infected with more than one strain [38]. Studies elsewhere indicate that the community is the main vehicle for MTB transmission [39–43]. However, this is contrary to our current results. Perhaps community transmission is more common in high TB incidence regions where chances of acquiring the infection outside are higher than inside the household [44]. This data shows that intra family and community TB transmission are important mechanisms of disease spread in this study.

Transmission of TB in children is a reflection of on-going transmission of MTB in a community [45]. This study used children as a proxy for transmission, to test the hypothesis that MTBC L4-U causes advanced lung disease and thus more transmissible [10–12, 25, 26, 46, 47] than other lineages circulating in peri-urban Kampala. Advanced lung disease was defined as the ability of MTBC lineage to cause extensive lung lesions and cavities ≤ 4 cm (extensive lung parenchymal damage) as described [24]. The data showed that the odds of causing advanced extent of lung disease by different MTBC lineages identified in this study area did not differ significantly (OR = 0.304, CI: 95 %; 0.039-2.326, P = 0.251) between MTBC lineages (Table 2). This lack of association remained even after adjusting for clinical and epidemiological characteristics of the children. These analyses suggest that MTBC lineages circulating in peri-urban Kampala are equally transmissible.

Limitations that of our study are that pediatric TB is a pauci-bacillary disease. This can lead to significant selection bias due to variability in culture of MTBC lineages. However, this was unlikely the case here as the distribution of MTB strains in children and adults was the same. Second, the samples size was limited and we therefore might have missed, true associations. Last, the inability of SNP markers to resolve the lineages furthers into strains, precluded us from defining actual chains of transmission. Nevertheless SNPs are robust markers in defining MTBC lineages.

## Conclusion

Our findings indicate that the majority of childhood TB is caused by MTBC Uganda family strains in Kampala, Uganda. There was no evidence that MTBC Uganda family differed in their propensity to transmit to children compared to other MTBC lineages.

## Abbreviations

MTBC: *Mycobacterium tuberculosis* complex
TB: Tuberculosis
SNP: Single nucleotide polymorphism
RT-PCR: Real time polymerase chain reaction
OR: Odds ratio
CI: Confidence interval
ELISA: Enzyme linked immunosorbent assay
HIV: Human immuno deficiency virus
TST: Tuberculin skin test
OADC: Oleic albumin dextrose catalase
BCG: Bacillus Calmette-Guerin
JCRC: Joint clinical research centre
DNA: Deoxyribonucleic acid
KC: Kawempe Community
MTB L4-U: *Mycobacterium tuberculosis* Uganda family
MTB L4-NU: *Mycobacterium tuberculosis* lineage 4 strains other than MTB Uganda family
MTB L4: *Mycobacterium tuberculosis* lineage 4 (Euro-American lineage)
MTB L3: *Mycobacterium tuberculosis* lineage 3 (East Africa India or Central Asian Stains)
WHO: World Health Organization

## References

[1] WHO (2012). Global tuberculosis report.

[2] WHO (2011). Global tuberculosis control report.

[3] Shingadia D, Novelli V (2003). Diagnosis and treatment of tuberculosis in children. Lancet Infect Dis.

[4] Swaminathan SRB (2010). Pediatric tuberculosis: global overview and challenges. Clin Infect Dis.

[5] Cantwell MFSZ, Costello AM, Sands L, Green WF, Ewing EP, Valway SE (1994). Brief report: congenital tuberculosis. N Engl J Med.

[6] Huang CC, Tchetgen ET, Becerra MC, Cohen T, Hughes KC, Zhang Z (2014). The effect of HIV-related immunosuppression on the risk of tuberculosis transmission to household contacts. Clin Infect Dis.

[7] Getahun H, Gunneberg C, Granich R, Nunn P (2010). HIV infection-associated tuberculosis: the epidemiology and the response. Clin Infect Dis.

[8] Malla B, Stucki D, Borrell S, Feldmann J, Maharjan B, Shrestha B (2012). First insights into the phylogenetic diversity of Mycobacterium tuberculosis in Nepal. PLoS One.

[9] Andrews JR, Noubary F, Walensky RP, Cerda R, Losina E, Horsburgh CR (2012). Risk of progression to active tuberculosis following reinfection with Mycobacterium tuberculosis. Clin Infect Dis.

[10] Aguilar D, Hanekom M, Mata D, van Pittius NC G, van Helden PD, Warren RM (2010). Mycobacterium tuberculosis strains with the Beijing genotype demonstrate variability in virulence associated with transmission. Tuberculosis (Edinb).

[11] Kato-Maeda M, Shanley CA, Ackart D, Jarlsberg LG, Shang S, Obregon-Henao A (2012). Beijing sublineages of Mycobacterium tuberculosis differ in pathogenicity in the guinea pig. Clin Vaccine Immunol.

[12] Rodrigo T, Cayla JA, Garcia De Olalla P, Galdos-Tanguis H, Jansa JM, Miranda P (1997). Characteristics of tuberculosis patients who generate secondary cases. Int J Tuberc Lung Dis.

[13] Wampande EM, Debanne SM, Asiimwe BB, Nsereko M, Mayanja H, Eisenach K (2013). Long-term dominance of Mycobacterium tuberculosis Uganda family in peri-urban Kampala-Uganda is not associated with cavitary disease. BMC Infect Dis.

[14] Asiimwe BB, Joloba ML, Ghebremichael S, Koivula T, Kateete DP, Katabazi FA (2009). DNA restriction fragment length polymorphism analysis of Mycobacterium tuberculosis isolates from HIV-seropositive and HIV-seronegative patients in Kampala, Uganda. BMC Infect Dis.

[15] Weis SE, Pogoda JM, Yang Z, Cave MD, Wallace C, Kelley M (2002). Transmission dynamics of tuberculosis in Tarrant county, Texas. Am J Respir Crit Care Med.

[16] Newton SM, Brent AJ, Anderson S, Whittaker E, Kampmann B (2008). Paediatric tuberculosis. Lancet Infect Dis.

[17] Nakaoka H, Lawson L, Squire SB, Coulter B, Ravn P, Brock I (2006). Risk for tuberculosis among children. Emerg Infect Dis.

[18] Moran-Mendoza O, Marion SA, Elwood K, Patrick D, FitzGerald JM (2010). Risk factors for developing tuberculosis: a 12-year follow-up of contacts of tuberculosis cases. Int J Tuberc Lung Dis.

[19] Coscolla M, Gagneux S (2014). Consequences of genomic diversity in Mycobacterium tuberculosis. Semin Immunol.

[20] Guwatudde D, Nakakeeto M, Jones-Lopez EC, Maganda A, Chiunda A, Mugerwa RD (2003). Tuberculosis in household contacts of infectious cases in Kampala, Uganda. Am J Epidemiol.

[21] Jaganath D, Zalwango S, Okware B, Nsereko M, Kisingo H, Malone L (2013). Contact investigation for active tuberculosis among child contacts in Uganda. Clin Infect Dis.

[22] CLSI (2008). Laboratory detection and identification of mycobacteria: approved guideline. CLSI document M48-A.

[23] Siddiqi SH, Hwangbo CC, Silcox V, Good RC, Snider DE, Middlebrook G (1984). Rapid radiometric methods to detect and differentiate Mycobacterium tuberculosis/M. bovis from other mycobacterial species. Am Rev Respir Dis.

[24] Falk AOCA, Pratt PC, Falk A, O’Connor AJB, Pratt PC, Webb JA, Weir JA, Wolinsky A (1969). Classification of pulmonary tuberculosis. Diagnosis standards and classifiaction of tuebrculosis.

[25] Lipsitch M, Nowak MA (1995). The evolution of virulence in sexually transmitted HIV/AIDS. J Theor Biol.

[26] Lipsitch M, Moxon ER (1997). Virulence and transmissibility of pathogens: what is the relationship?. Trends Microbiol.

[27] Perez-Velez CM (2012). Pediatric tuberculosis: new guidelines and recommendations. Curr Opin Pediatr.

[28] Perez-Velez CM, Marais BJ (2012). Tuberculosis in children. N Engl J Med.

[29] Chintu C, Mudenda V, Lucas S, Nunn A, Lishimpi K, Maswahu D (2002). Lung diseases at necropsy in African children dying from respiratory illnesses: a descriptive necropsy study. Lancet.

[30] Scott JA, Brooks WA, Peiris JS, Holtzman D, Mulholland EK (2008). Pneumonia research to reduce childhood mortality in the developing world. J Clin Invest.

[31] Morrison J, Pai M, Hopewell PC (2008). Tuberculosis and latent tuberculosis infection in close contacts of people with pulmonary tuberculosis in low-income and middle-income countries: a systematic review and meta-analysis. Lancet Infect Dis.

[32] WHO (2007). A research agenda for childhood tuberculosis: improving the management of childhood tuberculosis within national tuberculosis programmes; research priorities based on a literature review.

[33] Gessner BD, Weiss NS, Nolan CM (1998). Risk factors for pediatric tuberculosis infection and disease after household exposure to adult index cases in Alaska. J Pediatr.

[34] WHO: Global tuberculosis control-epidemiology, strategy, financing. http://who.int/tb/publications/global_report/2009/en/index.html 2009.

[35] Marais BJ, Hesseling AC, Gie RP, Schaaf HS, Beyers N (2006). The burden of childhood tuberculosis and the accuracy of community-based surveillance data. Int J Tuberc Lung Dis.

[36] Connell TG, Zar HJ, Nicol MP (2011). Advances in the diagnosis of pulmonary tuberculosis in HIV-infected and HIV-uninfected children. J Infect Dis.

[37] Nabyonga L, Kateete DP, Katabazi FA, Odong PR, Whalen CC, Dickman KR (2011). Determination of circulating Mycobacterium tuberculosis strains and transmission patterns among pulmonary TB patients in Kawempe municipality, Uganda, using MIRU-VNTR. BMC Res Notes.

[38] Dickman KR, Nabyonga L, Kateete DP, Katabazi FA, Asiimwe BB, Mayanja HK (2010). Detection of multiple strains of Mycobacterium tuberculosis using MIRU-VNTR in patients with pulmonary tuberculosis in Kampala, Uganda. BMC Infect Dis.

[39] Augustynowicz-Kopec EJT, Kozinska M, Kremer K, van Soolingen D, Bielecki J, Zwolska Z (2012). Transmission of tuberculosis within family-households. J Infect.

[40] Kasaie PAJ, Kelton WD, Dowdy DW (2013). Timing of tuberculosis transmission and the impact of household contact tracing. An agent-based simulation model. Am J Respir Crit Care Med.

[41] Verver S, Warren RM, Munch Z, Richardson M, van der Spuy GD, Borgdorff MW (2004). Proportion of tuberculosis transmission that takes place in households in a high-incidence area. Lancet.

[42] Wang PD, Lin RS (2000). Tuberculosis transmission in the family. J Infect.

[43] Wood R, Johnstone-Robertson S, Uys P, Hargrove J, Middelkoop K, Lawn SD (2010). Tuberculosis transmission to young children in a South African community: modeling household and community infection risks. Clin Infect Dis.

[44] Classen CN, Warren R, Richardson M, Hauman JH, Gie RP, Ellis JH (1999). Impact of social interactions in the community on the transmission of tuberculosis in a high incidence area. Thorax.

[45] Schaaf HS, Michaelis IA, Richardson M, Booysen CN, Gie RP, Warren R (2003). Adult-to-child transmission of tuberculosis: household or community contact?. Int J Tuberc Lung Dis.

[46] Brites D, Gagneux S (2011). Old and new selective pressures on Mycobacterium tuberculosis. Infect Genet Evol.

[47] Helke KL, Mankowski JL, Manabe YC (2006). Animal models of cavitation in pulmonary tuberculosis. Tuberculosis (Edinb).

